# Inhibition of Angiotensin II-Induced Cardiac Hypertrophy and Associated Ventricular Arrhythmias by a p21 Activated Kinase 1 Bioactive Peptide

**DOI:** 10.1371/journal.pone.0101974

**Published:** 2014-07-11

**Authors:** Rui Wang, Yanwen Wang, Wee K. Lin, Yanmin Zhang, Wei Liu, Kai Huang, Derek A. Terrar, R. John Solaro, Xin Wang, Yunbo Ke, Ming Lei

**Affiliations:** 1 Institute for Cardiovascular Diseases, Union Hospital, Huazhong University of Science and Technology, Wuhan, P. R. China; 2 Institute of Cardiovascular Sciences, Faculty of Medicine and Human Science, University of Manchester, Manchester, United Kingdom; 3 Department of Pharmacology, University of Oxford, Oxford, United Kingdom; 4 Department of Physiology and Biophysics, Center for Cardiovascular Research, College of Medicine, University of Illinois at Chicago, Chicago, Illinois, United States of America; 5 Faculty of Life Science, University of Manchester, Manchester, United Kingdom; Cleveland Clinic Lerner Research Institute, United States of America

## Abstract

Cardiac hypertrophy increases the risk of morbidity and mortality of cardiovascular disease and thus inhibiting such hypertrophy is beneficial. In the present study, we explored the effect of a bioactive peptide (PAP) on angiotensin II (Ang II)-induced hypertrophy and associated ventricular arrhythmias in *in vitro* and *in vivo* models. PAP enhances p21 activated kinase 1 (Pak1) activity by increasing the level of phosphorylated Pak1 in cultured neonatal rat ventricular myocytes (NRVMs). Such PAP-induced Pak1 activation is associated with a significant reduction of Ang II-induced hypertrophy in NRVMs and C57BL/6 mice, *in vitro* and *in vivo*, respectively. Furthermore, PAP antagonizes ventricular arrhythmias associated with Ang II-induced hypertrophy in mice. Its antiarrhythmic effect is likely to be involved in multiple mechanisms to affect both substrate and trigger of ventricular arrhythmogenesis. Thus our results suggest that Pak1 activation achieved by specific bioactive peptide represents a potential novel therapeutic strategy for cardiac hypertrophy and associated ventricular arrhythmias.

## Introduction

Cardiac hypertrophy (CH) is a critical intermediate step for the development of heart failure (HF) regardless of the inciting pathological stimulus. It is an independent risk factor in its own right in cardiovascular mortality and morbidity through interacting with other cardiovascular risk factors. Patients with CH and HF often experience fatal ventricular arrhythmias leading to sudden cardiac death resulting from a breakdown in heart rhythm, which underlies 50% of cardiovascular mortality. Controlling hypertrophic remodelling may therefore offer the most promising new therapeutic strategy for reducing cardiovascular morbidity and mortality in both CH and HF. Most existing therapies that have antihypertrophic effects target extracellular receptors in cardiac cells, but their effectiveness seems limited, and so attention has recently turned to the potential of targeting intracellular signalling pathways.[1]

Our work and the work of others over the past few years have led to the identification of new roles of an intracellular multifunctional signaling enzyme p21 activated kinase 1 (Pak1) in cardiac physiology, such as the regulation of cardiac ion channels and sarcomeric proteins in cardiac myocytes [2], [3]. Our studies of acute responses to active Pak1 revealed an anti-adrenergic activity related to activation of the protein phosphatase, PP2A, [2], [4], [5] resulting in enhanced myofilament response to Ca^2+^ [2] and a depressed response to adrenergically-mediated increases in heart rate and Ca^2+^ channel activity [3]. We also demonstrated that a significantly increased response to hypertrophic stress (chronic β-adrenergic stimulation, pressure overload) was observed in hearts of mice with Pak1-deficiency in cardiac tissue (Pak1^cko^) (Liu et al. 2011). Pak1^cko^ mice were vulnerable to cardiac hypertrophy and readily progress to cardiac failure under sustained pressure overload or pharmacological stress by Ang II or adrenergic agents [6], [7]. These observations indicate that Pak1 is a key regulator of acute and chronic cardiac function, and raise the possibility of activation of Pak1 as a new strategy for management of CH and other cardiac disorders. Application of FTY720 (a synthetic analogue structurally similar to sphingosine) induced Pak1 activation and restrained the development of CH in wild type mice with pressure overload stress, but not in Pak1-deficient mice (Pak1^cko^) mice with pressure overload stress, suggesting the anti-hypertrophic effect of FTY720 was likely due to its effect on activation of Pak1[6]. Thus these results suggest Pak1 as a potential novel anti-hypertrophic target for the treatment of CH and HF.

Here we report a bioactive peptide (PAP) derived from the Pak1 autoinhibitory region increases Pak1 activity, counteracts Ang II-induced pathological hypertrophy in *in vitro* and *in vivo* models and associated ventricular arrhythmias in *in vivo* models. Our data suggest that targeting Pak1 activation represents a novel therapeutic option for the management of cardiac hypertrophy and its associated ventricular arrhythmias.

## Methods

Animal studies were performed in accordance with the UK Home Office and institutional guidelines. The study and experimental protocols were approved by Manchester University Research Ethics Committee.

### Generation of PAP

PAP (Pak activating peptide) TSNSQKYMSFTDKSA was derived from the Pak1 autoinhibitory region and was linked to the 11-amino acid sequence YGRKKRRQRRR derived from HIV-1 trans-activating regulatory protein. The peptide YGRKKRRQRRRGTSNSQKYMSFTDKSA was synthesized in the proteomics core lab in Research Resource Center at University of Illinois at Chicago (UIC) and was confirmed by mass spectrometry.

### Neonatal rat cardiomyocytes hypertrophy

Neonatal rat cardiomyocytes (NRVMs) were isolated from 1–2 day old rats using a standard enzymatic method as we described previously [6]. Isolated NRVMs were plated onto laminin-coated coverslips. To examine the effect of PAP on angiotensin II (Ang II, Sigma-Aldrich) induced hypertrophy in NRVMs, NRVMs were treated with Ang II (500 nM) accompanied with or without PAP (20 µg/ml) for 48 h and NRVMs without treatment were taken for control. Thereafter, NRVMs were subjected to immunocytochemistry using the primary α-actinin antibody (1∶100, Sigma, A7811) and the secondary anti-mouse antibody conjugated to Alexa Fluoro. NRVMs were co-stained with DAPI to visualize their nuclei. Images of >150 visible cells were collected and their surface area measured using Image J software.

### Mouse cardiac hypertrophy

**Figure 1:**
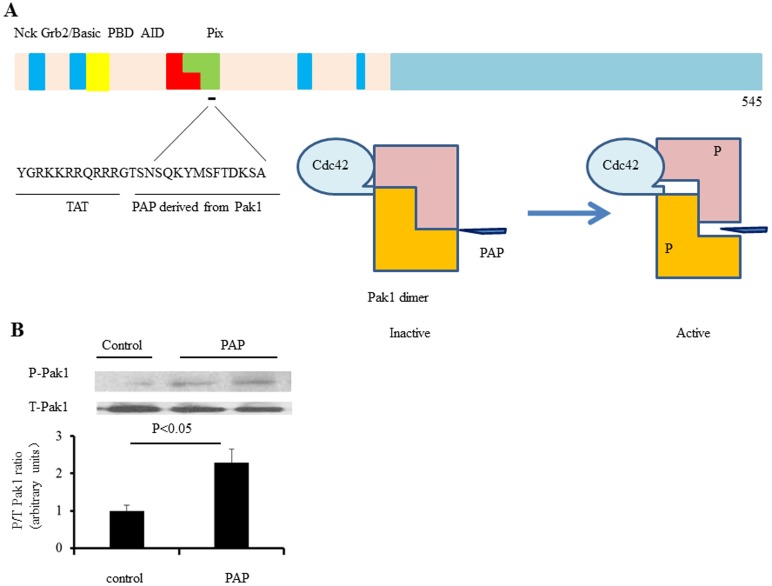
PAP and its interaction with Pak1. **A**: Pak1 is divided into N-terminal and C-terminal halves. The N-terminal half contains p21 binding domain (PBD) followed by a kinase inhibitory domain, which overlap with each other. The proline rich motifs interact with different cellular proteins including Nck, Grb2 and Pix, etc. PAP is derived from the autoinhibitory domain linked to a TAT sequence. PAP binds to Pak1 and may activate Pak1 through attenuation of Pak1 autoinhibition in a similar way as Cdc42 and Rac1 do. **B**: We examined the effects of the PAP on Pak1 phosphorylation in cultured neonatal rat ventricular myocytes (NRVMs) that were treated with PAP (20 µg/ml) for 2 hours. Immunoblotting analyses of Pak1 phosphorylation indicate that Pak1 activation was induced by angiotensin II (Ang II), in NRVMs (n = 3 independent experiments).

Cardiac hypertrophy was induced by administration of Ang II at 1 mg/kg/day for 7 days using osmotic mini-pumps (Alzet) implanted subcutaneously in 3-month-old male C57BL/6 mice. Figure 1 illustrates the experimental protocol for inducing hypertrophy, treatment and analysis. After the treatment, the heart weight (HW) and tibia length (TL) were measured and the HW/TL ratios were calculated to indicate cardiac hypertrophy. Animal studies were performed in accordance with the UK Home Office and institutional guidelines.

### Immunoblot analysis

Protein extracts (50 µg) were subjected to immunoblot analysis with antibodies against Pak1 (Cell signal, 1∶1000, Cell Signalling Technology Inc, Danvers, USA), phospho-Pak1 (2601, Thr 423, 1∶1000, Cell Signalling Technology Inc.). Immune-complexes were detected by enhanced chemiluminescence with anti-rabbit immunoglobulin G coupled to horseradish peroxidise as the secondary antibody (Abcam, Cambridge, UK).

### Immunohistochemical analysis

Ventricular tissue was frozen in OCT and 10 µm sections were collected using a cryostat. Sections were used for Masson's trichrome stain and Hematoxylin and eosin (HE) Staining; bright field images were taken for measuring cross-sectional area and fibrosis area. Approximately 150 randomly selected cardiomyocytes were measured to calculate the mean cross-sectional area and 30 randomly chosen frames of Masson's trichrome stained sections were quantified to assess the degree of myocardial fibrosis using Image J software (NIH, USA).

### Electrocardiography (ECG)

To monitor cardiac rhythm, we carried out *in vivo* ECG analysis on mice anesthetized with isoflurane (2.5%). RR interval, P wave duration, PR interval, QRS, JT and QT durations were recorded. Three-lead limb electrocardiographic (lead II) were recorded through subcutaneous needle electrodes using a Power lab 26 T system (AD Instruments, Hastings, UK). Signals were filtered (pass bandwidth: 50–500 Hz), digitized (16 bits, 2 kHz/channel), and analyzed (Chart v6.0 program, AD Instruments) to obtain signal-averaged ECGs. Recordings were carried out over 5 minutes to permit ECG recordings to stabilize and measurements of ECG parameters made from recordings obtained.

### Programmed electrical stimulation (PES)

Mice were killed by cervical dislocation (Schedule 1: UK Animals [Scientific Procedures] Act 1986). The heart was excised, cannulated and mounted onto a Langendorff system, then perfused (flow rate: 3 ml/min; Watson-Marlow Bredel Peristaltic pumps, model 505 S, Falmouth, Cornwall, UK) with Krebs' Ringer (KR) (mM)(NaCl: 119, NaHHCO_3_: 25, Glucose: 10, Na Pyruvate: 2, KCl: 4, MgCl: 1, KH2PO4:1.2, CaCl2) solution passed through 5 µm filter (Millipore, Watford, UK) warmed to 37°C using a water jacket and circulator (Techne model C-85A, Cambridge,UK). The heart was laid with its anterior surface facing up-ward immersed in KR solution in a warmed bath chamber thermally equilibrated with the myocardial perfusate for electrophysiological studies. To assess propensity to exhibit ventricular arrhythmias, Langendorff-perfused hearts from 3 to 4-month old mice were subjected to programmed electrical stimulation (PES) in which extra systolic S2 stimuli after successive trains of eight pacing S1 stimuli delivered at 8 Hz at S1S2 intervals, is progressively decremented by 1 ms with each successive pacing cycle. For burst pacing protocol, ventricular pacing with a train of 50 S1 at a cycle length of 20 ms at progressively increase in pacing current amplitude starting from basal threshold of ventricular capture until ventricular tachycardia or fibrillation was induced or current 35 mA was reached. Ventricular tachycardia was defined as six or more consecutive premature ventricular waveforms (tachycardia with regular waveforms defined as VT, while VF was characterized by irregular fibrillating waveforms).

### Epicardial activation mapping

An 8×8 multi-electrode array covering a 4 mm×4 mm area (electrodes were spaced by 0.55 mm) was used to record ventricular epicardial electrical mapping at a 3 kHz/channel sampling rate by covering the regions of the left ventricle. The arrays were connected through shielded leads to a 64-channel amplifier (SCXI-1102C, National Instruments Corporation Ltd., Newbury, UK). Acquired signals were continuously recorded to disk and displayed using custom-developed Labview 7.0 (National Instruments Corporation Ltd.) programs.

### Line scan confocal microscopy

Ventricular myocytes isolated from control, Ang II or Ang II plus PAP treated hearts were incubated with fluo-4 AM (10 µM) for 15 min. For Ca^2+^ transients recording, myocytes were electrically stimulated at 1 Hz by carbon-fibre electrodes placed at the side of the superfusion bath. Myocytes were imaged with a confocal microscope system that consisted of a Leica TCS NT scanning head coupled to a Leica DMIRB inverted microscope with a 100× oil immersion objective lens (1.2 NA, Leica) in line scan mode (2.6 ms per line). Excitation light (488 nm) was provided by an air-cooled 488 nm argon ion laser system (Uniphase Ltd, USA) and the emitted light was collected at wavelengths above 515 nm using a long-pass filter. For calcium spark recording, myocytes were incubated and imaged with similar method as above. Without external electrical stimulation, a quiescent myocyte was selected and an area of interest (defined by a single line placed across the cell longitudinally) was scanned repetitively. Both images were recorded using Leica TCS NT software and analysed using ImageJ software.

### Data Analysis

One-way ANOVA followed by post hoc testing was used for comparisons among multiple groups. Comparisons between 2 groups were performed using *Student's t* test. P values less than 0.05 are considered statistically significant. Data are presented as mean ± SEM.

## Results

### PAP enhances Pak1 activity in cardiac myocytes

Figure 1A describes the strategy of the design of PAP. PAP is derived from the Pak1 kinase inhibitory domain (KID) linked to a TAT sequence. It binds to the Pak1 molecule to dislocate the same endogenous sequence that is involved in formation of Pak1 autoinhibition. Therefore it may alter Pak1 activity through attenuation of Pak1 autoinhibition in a similar way as Cdc42 and Rac1 do. Thus, although PAP is derived from the Pak1 autoinhibitory region, its effect on Pak1 activities could be stimulatory, instead of inhibitory. We tested this hypothesis by examining the effect of the PAP on Pak1 phosphorylation in cultured neonatal rat ventricular myocytes (NRVMs) that were treated with PAP (20 µg/ml) for 2 hours. As shown in Figure 1B, after PAP treatment, phosphorylated Pak1 was significantly increased in these primary cardiac myocytes characterized by Western blots (three independent experiments p<0.05), suggesting that PAP was able to enhance Pak1 phosphorylation as we predicted and therefore increased rather than inhibited Pak1 activity in cardiac cells.

### PAP abrogates Ang-II induced hypertrophy in *in vitro* and *in vivo* models

**Figure 2:**
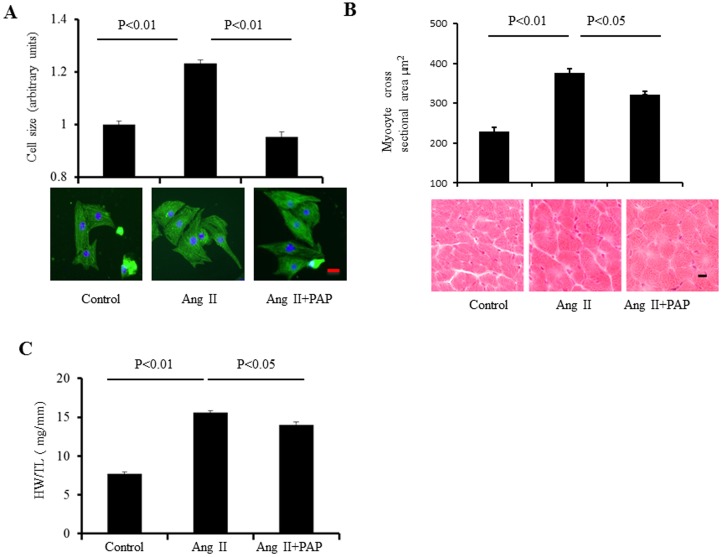
PAP abrogates Ang-II induced hypertrophy in *in vitro* and *in vivo* models. **A**: NRVMs were treated with Ang II (500 nM) with or without co-treatment of PAP (20 µg/ml) for 48 h, followed by α-actinin immunostaining. Cell size was measured and presented as the bar graphs (upper panel, 450 cells from three independent experiments). Representative images of double staining of NRVMs are shown in lower panel (green staining for α-actin; blue for DAPI, scale bar: 20 µm). **B**: Mean of cross-sectional areas measurements (upper panel); HE staining of heart cross-sections (lower panel, scale bar: 20 µm, n = 4). **C**: HW/TL ratios of mice before and after Ang II or Ang II+PAP treatments (n = 4).

The consequences of Pak1 activation by PAP on Ang-II induced hypertrophy were then investigated in both *in vitro* primary cardiac myocytes and *in vivo* mouse models. Firstly, such an effect was examined in cultured neonatal rat ventricular myocytes (NRVMs). Cell hypertrophy was induced by treating these cells with Ang II (500 nM) for 24-to 48 h. To determine the effect of PAP on Ang II induced hypertrophy, the NRVMs were treated with Ang II (500 nM) without or with PAP (20 µg/ml) treatment for 48 h. As shown in Figure 2A and 2B, NRVMs treated with both Ang II and PAP showed less hypertrophy characterized by cell size than NRVMs treated by Ang II without PAP (0.95±0.02 in Ang II+PAP group vs. 1.23±0.01 in Ang II treated group). The cell sizes were normalized to those of the control group (n≈450 cells from 3 independent experiments, p<0.01). These results suggest that PAP abrogated Ang II-induced hypertrophy in *in vitro* cardiac myocyte model. Secondly, such an anti-hypertrophic effect of PAP was further examined under in-vivo conditions in male C57BL/6 mice. Animals were divided into three experimental groups: mice treated with purified H_2_O (as the control group), mice treated with Ang II (1 mg/kg/day, defined as Ang II group) without or with PAP co-treatment (1 mg/kg/day, defined as Ang II + PAP group). All substances were delivered by the osmotic mini-pumps for a period of 7 days. CH was characterized by measuring the heart weight/tibia length (HW/TL) ratios and cell mean cross-sectional areas. Ang II treatment induced a remarkable ventricular hypertrophy indicated by substantially increased (HW/TL) ratios (7.7±0.3 mg/mm in control group (n = 6) vs. 15.6±0.2 mg/mm in Ang II group, p<0.01, n = 6) and mean cross-sectional areas (228±10 µm^2^ in control group (n = 6) vs. 376±11 µm^2^ in Ang II group (n = 6), p<0.05 Figure 2.B,C). However, such Ang II-induced hypertrophy was significantly inhibited by PAP in Ang II + PAP group as shown in Figure 2C.

### PAP attenuates alteration in ventricular electrophysiological properties and arrhythmias associated with Ang II-induced hypertrophy

Hypertrophy creates arrhythmic substrates in ventricle on which the transient factors (so called triggers) operate to initiate a ventricular tachyarrhythmia [8]. Therefore, any agents limiting hypertrophic remodeling could be potentially anti-arrhythmic in this setting. We thus further investigated the effects of PAP on ventricular electrophysiological properties and ventricular arrhythmogenesis associated with Ang II-induced hypertrophy.

**Figure 3:**
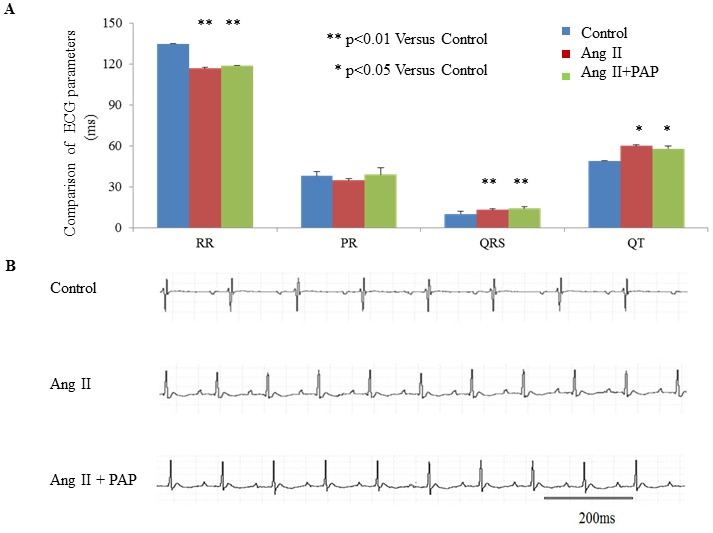
PAP protects heart from ventricular arrhythmia associated with hypertrophy. **A**: Comparison of *in vivo* electrocardiographic parameters between before and after treatment of Ang II only or Ang II plus PAP treatment for 7 days, (n = 4–6 per group. **B**: Representative recordings of typical *in vivo* ECG recordings from anesthetized mice before and after Ang II treatment (1 mg/kg/day) with or without co-treatment of PAP (1 mg/kg/day) for 7 days.

Firstly, surface ECG recording was performed on anesthetized C57BL/6 mice before and after the operation of insertion of osmotic mini-pump for delivering of Ang II, PAP or control H_2_O. The same experimental groups were employed as in the hypertrophy-inducing experiments described in Figure 2. As shown in Figure 3, Ang II significantly increased heart rate (HR), but prolonged QRS and QT intervals (Panel A), suggesting that Ang II led to cardiac electrical system remodeling. There were no significant differences in HR, QRS and QT intervals between Ang II and Ang II+PAP groups. Ang II+PAP group displayed less decrease in RR interval than Ang II group, which indicates that PAP may partially blunt the effect of Ang II on cardiac electrical properties under *in vivo* conditions.

**Figure 4:**
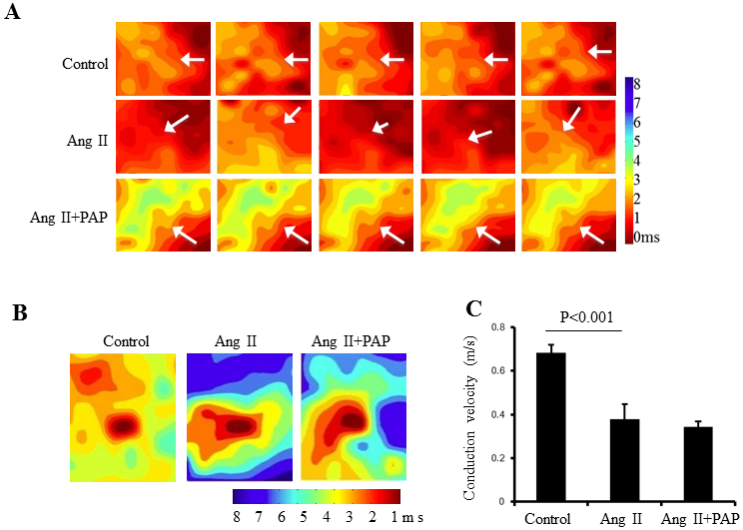
Ventricular epicardial electrical mapping with a multi-electrode array (MEA). **A**: Representative activation maps of five successive cardiac cycles under sinus rhythm obtained from the hearts from the control group and Ang II+PAP group showed a general pattern of sequential activation, whereas the hearts from Ang II group often showed a disordered pattern with beat-to-beat variations (n = 6), inserted arrows indicate the conduction direction. **B**: Pacing induced activation maps generated by pacing in the center of array on the epicardium of left ventricle from mice without any treatment and treated with Ang II or Ang II + PAP (n = 6 for each group). **C**: Comparison of left ventricular conduction velocity (n = 6 for each group).

Secondly, we examined the effect of PAP on Ang II-induced ventricular electrical remodeling associated with hypertrophy in mice under *ex vivo* conditions. We characterized the left ventricular epicardial conduction properties, which were studied by epicardial electrical mapping using a multi-electrode array (MEA) under conditions of either sinus rhythm or regular pacing by programmed electrical stimulation (PES) on isolated Langendorff-perfused hearts. Representative examples of activation maps in five successive cardiac cycles at sinus rhythm are shown in Figure 4A. Activation maps obtained from the hearts from the control group and Ang II+PAP group showed a general pattern of sequential activation, whereas the hearts from Ang II group often showed a disordered pattern with beat-to-beat variations. Similar observations were consistently made in 4–6 hearts in each group. Such MEA recordings also permitted determinations of conduction velocities under PES condition as shown in Figure 4B. Isochronal maps thus illustrated a characteristically slower conduction in both Ang II and Ang II+PAP groups in contrast to that shown by the control group (Fig 4B and C).

**Figure 5:**
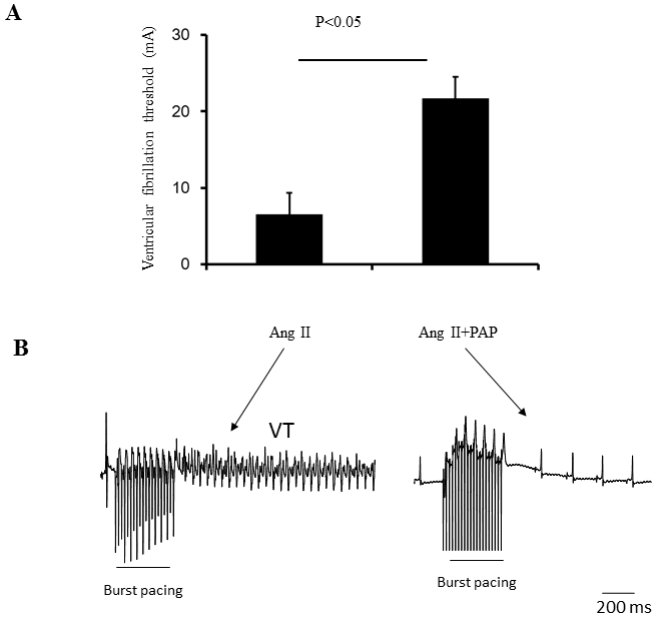
Conduction velocity under PES condition. **A**: Ventricular fibrillation threshold of the heart from mice treated with Ang II only and Ang II + PAP. **B**: Representative examples of ECG recordings from mice subjected to ex-vivo S1S1 ventricular pacing at 6.5 mA amplitude. Episodes of arrhythmias were showed in mice treated with Ang II only (n = 4 per group).

Thirdly, the possible phenotypic effect of PAP induced Pak1 activation on ventricular tachyarrhythmic tendency was also investigated in *ex vivo* mouse hearts following Ang II-induced hypertrophy (Figure 5). The mice were subjected to programmed electrical pacing (PES) using the Langendorff perfusion system. The presence and the frequency of arrhythmias were first compared using PES in which extra systolic S2 stimuli after successive trains of eight pacing S1 stimuli delivered at 8 Hz at S1S2 intervals, is progressively decremented by 1 ms with each successive pacing cycle. The protocol was terminated when hearts reached ventricular effective period (VERP) or went into arrhythmias. One of four animals studied in Ang II group, but no animals in the control or Ang II + PAP groups (four from each group), showed spontaneous or pacing induced VT. There was no significant difference in VERP between three groups. It was possible to assess arrhythmic thresholds in Langendorff-perfused hearts subject to progressively increased step current stimuli expressed normalized to their threshold stimuli until an endpoint of VT or VF. As shown in Figure 5, mice treated with Ang II alone showed significant a higher susceptibility of VT/VF that is characterized as remarkably lower pacing threshold leading to ventricular tachycardia or fibrillation (6.5±2.9 mA) compared with the mice co-treated with Ang II and PAP (21.6±3.8 mA, p<0.05), which suggests PAP prevents ventricular arrhythmogenesis in Ang II induced hypertrophic mice.

**Figure 6:**
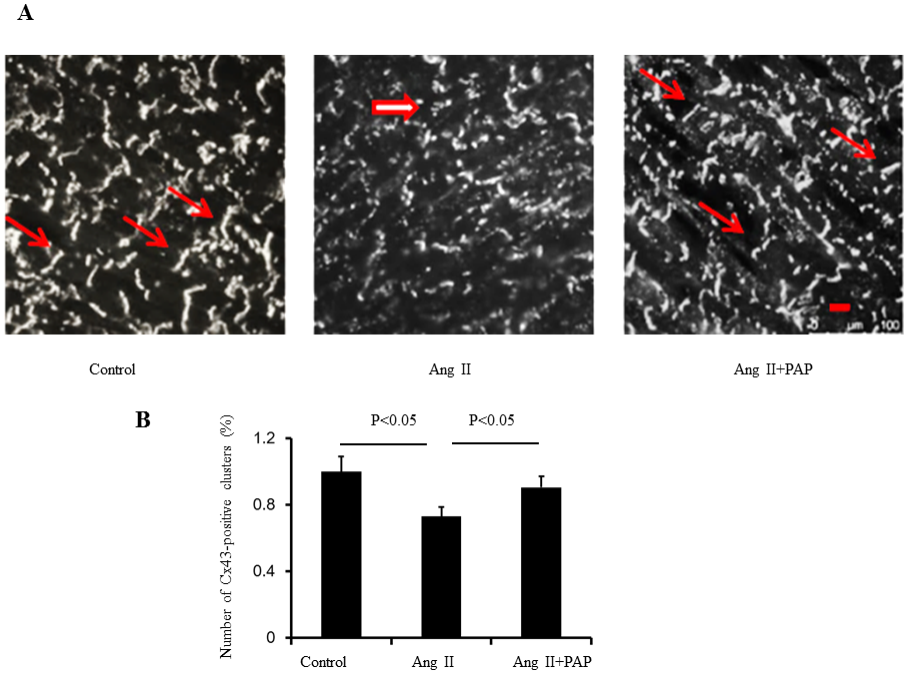
PAP treatment ameliorated Ang ll-induced decrement and spatially heterogeneous distribution of Cx43. **A**: Representative images of Cx43 staining. Thick arrows point to diffuse Cx43 labeling in the cytoplasm, whereas thin arrows show Cx43 distributed in intercalated discs. **B**: Immunostaining of Cx 43 was performed on sections of the heart from mice treated with Ang II or Ang II + PAP. The number of Cx43-positive clusters of Cx43 labeling are quantified, as shown in the bar graphs.(n = 4 per group,Scale bar, 20 um)

Remodeling in tissue structure in chronic forms of heart disease conditions such as CH leads to changes in expression and distribution patterns of gap junctions that is likely to alter the conduction properties of myocardium and contributes to arrhythmogenesis, independent of changes in the active membrane properties of individual cells. Both in experimental animals and in humans, prolonged hemodynamic overload is more commonly associated with significant downregulation of Cx43 expression, as well as lateralization of gap junctional protein away from the intercalated disks, i.e., with gap junction remodeling (GJR). [9]–[11]. In the final series of experiments, we investigated the expression and distribution patterns of major ventricular gap junction proteins Cx43 in left ventricular tissue from mouse hearts of three experimenting groups. Immunostaining of Cx43 was performed on sections of the hearts from mice without treatment (control group), treated with Ang II or Ang II+PAP. The number of Cx43-positive clusters of Cx43 labeling are quantified and expressed by the bar graphs (n = 4 hearts per group). As shown in Figure 6, PAP treatment significantly ameliorated the Ang II-induced alteration in both the expression and the distribution pattern of Cx43. This suggests the anti-arrhythmic effect of PAP in Ang II induced mouse hypertrophic model is at least partially due its effect on Ang II-induced Cx43 remodeling.

### PAP attenuates Ang II-induced hypertrophic associated Ca^2+^ sparks, waves and dyssynchronous Ca^2+^ transients

**Figure 7:**
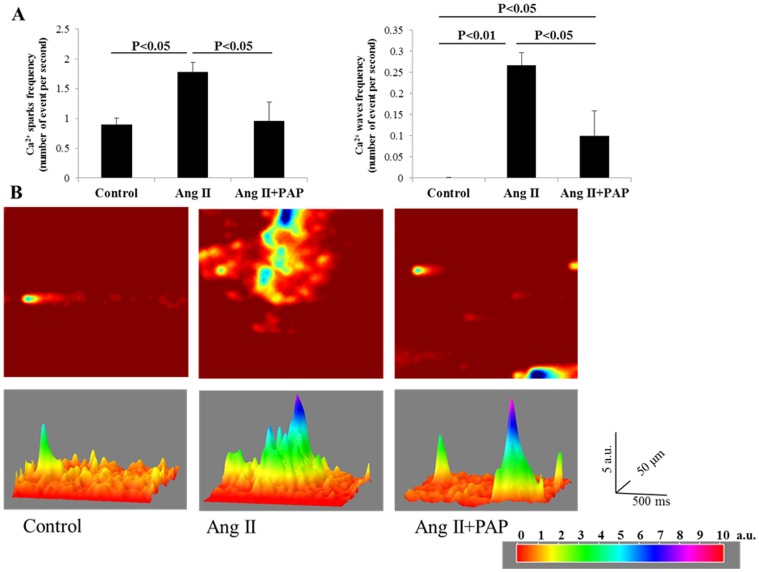
PAP reduced Ang II induced increase in frequencies of calcium sparks and waves in ventricular myocytes. **A**: The frequencies of calcium sparks (left panel) and waves (right panel) were measured from single ventricular myocytes isolated from mice administered with Ang II (10 mg/kg/day) with or without co-treatment of PAP (1 mg/kg/day) delivered by minipump for 7 days. The measurements were presented as means ± S.E.M (Control: n = 13; Ang II: n = 15; Ang II+PAP: n = 18). **B**: The 2D and 3D representative images showing calcium sparks and waves. Note: 2D Images were adjusted to enhance the contrast.

Abnormal intracellular Ca^2+^ ([Ca^2+^]_i_) handling has been attributed as a major cellular mechanism underlying ventricular arrhythmias associated with CH and HF [12]. We next assessed whether PAP is able to attenuates abnormal intracellular Ca^2+^ ([Ca^2+^]_i_) events in hypertrophied myocytes induced by Ang II. Firstly, spontaneous calcium sparks and waves (Figure 7) were measured in quiescent ventricular myocytes isolated from hearts treated with Ang II (10 mg/kg/day), or Ang II (10 mg/kg/day)+PAP (1 mg/kg/day) or H_2_O (control) for 7 days. As shown in Figure 7A, the frequencies of calcium sparks and waves (upper panel) of Ang II group (Sparks: 1.78±0.31/s; waves: 0.27±0.06/s) were significantly increased compared with control group (sparks: 0.90±0.11/s, p = 0.018; waves: 0.00±0.00/s, p = 0.0003) and Ang II+PAP group (sparks: 1.16±0.23/s, p = 0.020; waves: 0.10±0.03/s, p = 0.013), in other words, Ang II+PAP group displayed a significant lower in frequencies of calcium sparks and waves compared with Ang II group, which indicates that PAP blunted the effect of Ang II induced increase in frequencies in occurrence of spontaneous calcium sparks and waves. The representative 2D and 3D images shown in Figure 7B indicated the increased occurrences of calcium sparks and waves in Ang II treated myocytes and abated occurrences of calcium sparks and waves in Ang II+PAP treated myocytes.

**Figure 8:**
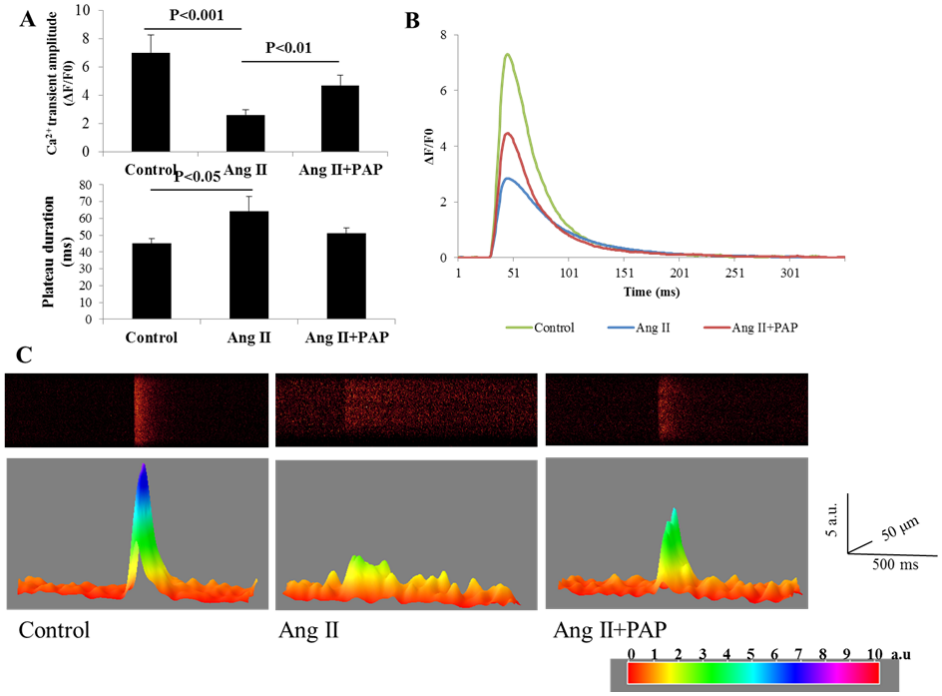
PAP restored the Ang II induced reduction of amplitudes and prolongation in peak-plateau of calcium transients in ventricular myocytes. **A**: The amplitude of the peak of calcium transients (upper panel) was measured by ΔF/F0. The duration of peak-plateau phase of the calcium transients (lower panel) was measured as the time interval between the upstroke of the fluorescence signal (measured at 80% of the maximum value) and the corresponding point on the decay (also measured at 80% of the maximum value). Both were presented as mean ± S.E.M (Control: n = 14; Ang II: n = 18; Ang II+PAP: n = 11). **B**: The representative traces showing the calcium transients of each group. C: The 2D and 3D representative images showing calcium transients.

Secondly, calcium transients (Figure 8) were measured in paced myocytes with field stimulation at 1 Hz. The calcium transients were recorded and normalised as ΔF/F0 as shown in Figure 8. In the upper panel of Figure 8A, the amplitudes of calcium transients of Ang II-treated myocytes (2.60±0.36) were significantly reduced compared with control group (7.00±1.27, p = 0.001), and the amplitudes of calcium transients were significantly recovered in Ang II+PAP-treated cardiomyocytes (4.70±0.70, p = 0.006). Furthermore, as shown in Figure 8A, the duration of peak-plateau phase of the calcium transients (lower panel) was measured as the time interval between the upstroke of the fluorescence signal (measured at 80% of the maximum value) and the corresponding point on the decay (also measured at 80% of the maximum value). The peak-plateau duration is significantly prolonged in Ang II-treated cardiomyocytes (64.42±8.51 ms, p = 0.032), compared with control group (45.13±2.87 ms), while peak-plateau duration in Ang II+PAP treated group is significant shorter (51.05±3.39 ms) than Ang II-treated cardiomyocytes despite is still longer than control group. Figure 8B showed the representative traces of the calcium transients generated by computer software. Figure 8C showed 2D and 3D representative examples of calcium transients.

## Discussion

In the present study, we explored the effects of a Pak1 bioactive peptide PAP on Ang II induced hypertrophy and associated ventricular arrhythmias in *in vitro* and *in vivo* models. Our data demonstrate that Pak1 activation by PAP is able to attenuate ventricular hypertrophic remodeling and associated ventricular arrhythmias induced by Ang II. Our findings suggest that Pak1 activation offers a novel therapeutic strategy for management of cardiac hypertrophy and its associated arrhythmias.

### PAP as a Pak1 activator

The study began by developing a specific Pak1 activating peptide. We took an advantage of a previous reported peptide (KI, PID) targeting at Pak1[13], [14]. Although PAP (KI, PID) was initially reported as a Pak1 inhibitor [13], [14], subsequent studies [15], [16]and ours (Figure 1) indicate that PAP actually increased Pak1 activity.

PAP activated Pak1 in cardiomyocytes (Figure 1). This is consistent with earlier observations that PAP produces the same cytoskeletal effects as Pak1 activation. The peptide reduced paxillin density at the cell periphery [15]. The same phenotype was observed in mammalian cells expressing constitutively active Pak1 [16]. In another study [17], PAP (KID) induced cell cycle arrest. Interestingly, the inhibitory effects of PAP on cell cycle progression was not blocked or reversed by expression of constitutively active Pak1 [17], suggesting that the peptide activated, instead of inhibited Pak1. Thus PAP is Pak1 bioactive peptide.

### Antihypertrophic effect of PAP

Since hypertrophy is regarded both as an intermediate step in and a determinant of HF, the discovery of molecular, cellular mechanisms and their signaling pathways underlying hypertrophic remodeling and the identification of potential therapeutic approaches for treating HF are of paramount importance. Although many signal transduction cascades have been demonstrated as important regulators to facilitate the induction of cardiac hypertrophy, the signaling pathways for suppressing hypertrophic remodeling remain largely unexplored. Our recent studies have revealed the negative effect of Pak1 on the development of pathological hypertrophy. Our study using primary cardiomyocytes and cardiomyocyte-specific Pak1 knockouts (Pak1^cko^) revealed an anti-hypertrophic effect of Pak1 [6]. In NRVMs, overexpression of constitutively active Pak1 attenuated phenylephrine-induced hypertrophic responses, whereas knockdown of Pak1 in NRVMs caused a greater hypertrophy after phenylephrine stimulation. This anti-hypertrophic property of Pak1 was further substantiated by the study of Pak1^cko^ mice. The Pak1^cko^ mice showed cardiac hypertrophy that was greater than in controls following two weeks of pressure overload, and also showed a rapid progression to heart failure after five weeks of load stress.[6] The Pak1^cko^ mice also demonstrated enhanced hypertrophy in response to angiotensin II infusion. Furthermore, application of FTY720 (a synthetic analog structurally similar to sphingosine) induced Pak1 activation and restrained the development of cardiac hypertrophy wild-type mice stressed by a pressure overload, but not in Pak1^cko^ mice, suggesting the anti-hypertrophic effect of FTY720 was likely due to its function on activation of Pak1 [6].

In line with the previous work, in the present study, we demonstrated a significant antihypertrophic effect of PAP that is able to activate Pak1. In the *in vitro* condition, PAP completely inhibited Ang II-induced cell hypertrophy in NRVMs (Figure 2A), which demonstrates the inhibitory effectiveness of activation of Pak1 by PAP on Ang II-induced cardiac cell hypertrophy. However, with *in vivo* conditions, we noted that PAP only partially inhibited Ang II effects in mouse ventricle (Figure 2B and C). This difference in effects of *in vitro* versus *in vivo* conditions could be due to the molecular size or dosage of PAP, and that the necessity for PAP to pass through cell membranes to interact with Pak1. The peptide may also be more susceptible to protease degradation before and after entering cardiac cells.

**Figure 9:**
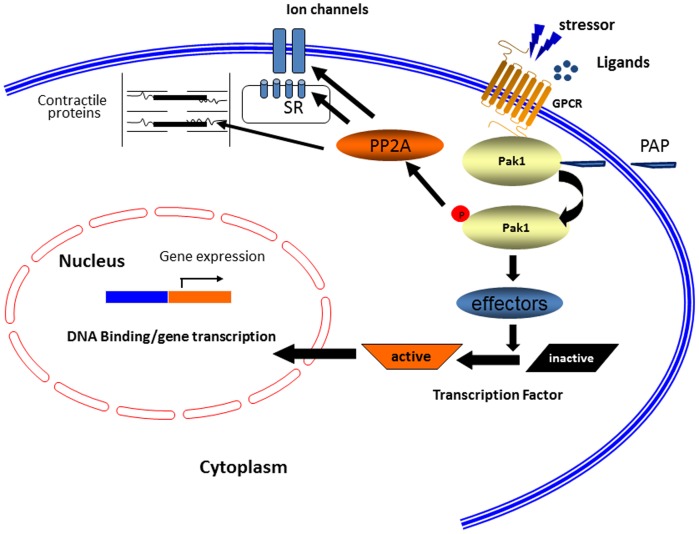
Regulation of cardiac excitation and hypertrophy by Pak1. Pak1 regulates the activities of ion channels, Ca^2+^ handling and myofilament proteins through PP2A. Cardiac hypertrophy induced under pathological conditions is suppressed by Pak1 through JNK signaling and regulation of Cx43 expression and distribution.

How does PAP associated Pak1 activation lead to antihypertrophy? This is likely to be through Pak1 action on JNK signaling as we demonstrated recently. As illustrated in Figure 9, Pak1 activates another kinase called JNK (c-Jun N-terminal kinase), which in turn phosphorylates and inactivates a transcription factor called NFAT, which is essential for activation of the hypertrophic genes such as atrial natriuretic peptide (ANP), brain natriuretic peptide (BNP). Thus, the activation of Pak1 would lead to activation of this JNK signaling cascade and a downregulation of NFAT.

### Antiarrhythmic effect and underlying mechanistic action of PAP

Patients with CH and HF often suffer arrhythmias resulting from a breakdown in the control of cell membrane excitability, potentially leading to sudden cardiac death which underlies 50% of the cardiovascular mortality.[18] Conversely, therapies that induce regression of hypertrophy decrease the risk of these cardiovascular events including ventricular arrhythmias independent of reductions in the remaining cardiovascular risk factors.[18] Development of effectively targeted anti-arrhythmic agents for the treatment of malignant cardiac arrhythmias, ventricular tachyarrhythmias in particular, associated with CH and HF remains a major challenge despite huge efforts that have been made over the past few decades. Our study indicates that activation of Pak1 could be a potential therapeutic strategy for prevention/inhibition of ventricular tachycardiarrhythmias associated with CH and HF. Thus mice co-treated with Ang II and PAP were less susceptible to pacing induced ventricular arrhythmias than those treated with Ang II alone, indicating the antagonizing effect of PAP on Ang II induced ventricular electrical remodelling and associated ventricular arrhythmias.

The inhibitory effect of PAP on Ang II-induced hypertrophy associated ventricular arrhythmogenesis is likely at least partially due to its effect on abnormal Ca^2+^-handling in hypertrophied myocytes. It is known that cardiac myocyte function is dependent on the synchronized movements of Ca^2+^ into and out of the cell, as well as between the cytosol and sarcoplasmic reticulum (SR). These movements determine cardiac rhythm and is mediated by a number of critical Ca^2+^-handling proteins and transporters including L-type Ca^2+^ channels (LTCCs), sodium/calcium exchangers in the sarcolemma, and sarcoplasmic reticulum (SR) calcium ATPase 2a (SERCA2a), ryanodine receptors, and cardiac phospholamban in the SR. Increased SR Ca^2+^ leak during diastole as a result of RyR2 dysfunction is a hallmark of cardiac hypertrophy and HF and serves as a major mechanism of rhythm disturbance in these conditions. As shown in Figure 7 the frequencies of spontaneous calcium sparks and waves measured in quiescent ventricular myocytes of Ang II group were significantly increased compared with control group, indicating increased SR Ca^2+^ leak due to RyR2 dysfunction in these myocytes. The dyssynchrony of Ca^2+^ transients is also demonstrated in calcium transients (Figure 8) were measured in paced myocytes. Thus, the amplitudes of calcium transients of Ang II-treated myocytes were significantly reduced compared with control group, furthermore, the peak-plateau duration is also significantly prolonged in Ang II-treated cardiomyocytes. In contrast, Ang II + PAP treated myocytes displayed a significant lower in frequencies of calcium sparks and waves in in quiescent ventricular myocytes compared with Ang II group, which indicates that PAP blunted the effect of Ang II induced increase in abnormal Ca^2+^-handling in hypertrophied myocytes. Improvements in knowledge of Ca^2+^ dynamics in health and disease have led to an increased understanding of the therapeutic potential of targeting Ca^2+^-handling proteins.

On the other hand, the regulation of Pak1 on Cx43 may also play an important role in inhibitory effect of PAP on Ang II-induced hypertrophy associated ventricular arrhythmogenesis. In our previous studies with Ai et al, we demonstrated that Pak1 induces dephosphorylation and reduction of activities of Cx43 as demonstrated in dye coupling through activation of phosphatase PP2A in isolated ventricular myocytes. On the other hand, Pak1 increases expression of Cx43 significantly [19]. Therefore, there is a balance between two effects produced by Pak1. In the models we employed, Pak1 PAP increases expression of Cx43 in the presence of ANG II or the peptide antagonizes the ANG II effect on reduction of Cx43 expression. The observation further confirmed our hypothesis that the peptide is a Pak1 activator as it produces the same effect on Cx43 expression as the constitutively active Pak1 does. A reduction of Cx43 expression induced by ANG II not only may lead to reduced Cx43 activities, it may be compounded by severe imbalance of local activities of Cx43, which is arrhythmogenic ([20]. Therefore, activation of Pak1 by PAP have anti-arrhythmic effects and such effects are thought multiple signaling mechanisms.

In conclusion, the results reported here suggest that a Pak1 bioactive peptide, PAP antagonizes Ang II effects in the heart. Such protective effects are likely mediated through multiple mechanisms. As illustrated in Figure 9, PAP treatment leads to the activation of Pak1, and through transcriptional mechanisms, Pak1 inhibits hypertrophic remodelling of the heart. Such chronic effects would modulate the arrhythmic “substrate” in the Ang II hypertrophic model. On the other hand, as illustrated in Figure 9, PAP induced Pak1 activation may also lead to a number of acute modifications of the function and activity of ion channels and Ca^2+^ handling proteins, particularly through altering their phosphorylation states, and may therefore counterbalance intracellular adrenergic signalling. Both are likely through PP2A dependent regulation, such effect would act on the “triggers” of ventricular arrhthmogenesis in Ang II hypertrophic model.

Our approach by using PAP has advantages over traditional drug administration, including the potential for high specificity and low toxicity. Since Ang II is an important mediator in cardiac remodeling and associated ventricular arrhythmias following myocardial infarction, it stimulates the progression of CH and HF, activation of Pak1 may thus represent a novel cardioprotection strategy in these clinical settings.
